# High sensitivity refractive index sensing using zone plate metasurfaces with a conical phase profile

**DOI:** 10.1038/s41598-022-12849-3

**Published:** 2022-05-28

**Authors:** Manar Abdel-Galil, Mohamed Swillam, Yehea Ismail, Diaa Khalil

**Affiliations:** 1 Department of Electrical and Communications Engineering, Faculty of Engineering, Ain Shams University, Cairo, Egypt; 2 Center for Nanoelectronics and Devices, American University in Cairo/Zewail City of Science and Technology, Cairo, Egypt; 3 Department of Physics, School of Science and Engineering, American University in Cairo, New Cairo, 11835 Egypt

**Keywords:** Optics and photonics, Applied optics, Optical materials and structures

## Abstract

In this paper, we showed how a bulky Axicon lens can be transformed to a compact binary zone plate with conical phase profile. We built three zone plates made of three different materials and designed each zone plate to be used in high sensitivity refractive index sensing. This work is complementary to another work we have done before in which we showed mathematically how maximum sensitivity can be achieved in case of using an Axicon lens in sensing. The zone plates are designed to generate a Bessel–Gauss beam at the wavelength of 3.3 microns at which the absorption of methane gas is maximum leading to a maximum change in the refractive index. The generated intensity in the output is very sensitive to any slight change in the refractive index of the surrounding medium. Therefore, if an optical detector is positioned at the point of maximum change in the intensity with refractive index, we can easily measure the change in refractive index and hence the percentage of the gas with very high sensitivity.

## Introduction

A Bessel beam can be viewed as the optical Fourier transform of a ring and this is how Durnin et al. arrived to an experimental approximation to the mathematical Bessel beam^1^. There are two main methods in the literature that are used to generate a Bessel beam. In the first method^1,2^, an annular ring (an annular slit) is placed at the focal plane before a convex lens. However, this initial method of Bessel beam generation was inefficient as most of the power from the Gaussian beam is blocked by the annular slit^3^. The second main method of generating a Bessel beam is using an axicon or a conical lens^4^. Using an axicon is much more efficient than using an annular slit because the axicon allows most or all of the Gaussian beam input to transmit through it. Other methods are also used to generate Bessel beams such as the use of holographic techniques or the use of a Fabry–Perot cavity together with an annular aperture^1^.

An axicon lens is a type of optical element that was first introduced in 1954 by McLeod^5^. Unlike the usual lenses, axicons do not have an exact one focus but rather a range of foci forming a focal line and therefore, we cannot define a certain focal length for this type of lense. However in our structures in this paper, we are going to define a parameter $f$ that represents the midpoint of the focal range. Axicon lenses have been employed in various applications such as laser machining^6,7^, corneal surgery^8^, imaging with an extended depth of focus^9^ and others^10–17^. Axicons can be classified into three categories: reflective^2,8,18–27^, refractive^28,29^ and diffractive^2,22^. In a previous work^30^, we showed how high sensitivity refractive index sensing can be attained using refractive Axicon lenses. We derived an analytical expression for the sensitivity and the condition for the Axicon base angle that leads to the maximum sensitivity. We also showed how we can practically choose this angle to maximize the practical sensitivity^30^. In this work, we explain how a bulky refractive Axicon lens can be converted to its more compact version of the binary zone plate lens while preserving its conical phase profile. Following that, we design three zone plate lenses at 3.3 microns which is the wavelength at which the absorption of the methane gas is maximum and thus a maximum change in the refractive index due to methane is also expected. The three zone plates we designed use the condition of maximum sensitivity in Ref.^30^ and thus attain high sensitivity of refractive index sensing.

## From bulky Axicon to compact binary zone plate

**Figure 1:**
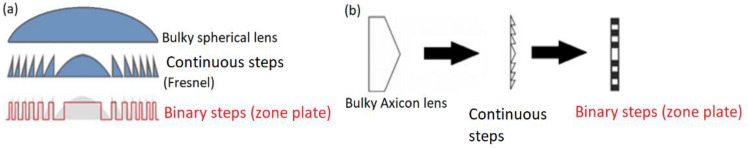
(**a**) Transforming a bulky spherical lens to either continuous steps (Fresnel) or binary steps (zone plate). (**b**) Transforming a bulky conical Axicon lens to either continuous steps or binary steps (zone plate).

To convert a conventional bulky spherical lens to a metasurface lens, we can either remove the extra dielectric material to get a Fresnel lens with continuous steps or we can discretize the steps into discrete binary steps to get a binary lens with only two levels which is called a zone plate lens^31^ as shown in Fig. 1a.

We follow the same analogy to convert a bulky Axicon lens into a binary zone plate metasurface as illustrated in Fig. 1b. In order to design a zone plate metasurface with conical phase profile, we should find the widths of the binary zones (both opaque and transparent zones). In other words, we need to find the positions of the zone boundaries in order to completely identify the designed zone plate. In the following sections, we present the conical phase profile of the bulky conical Axicon lens and we show how it could be used to find the positions of the zone boundaries in case of the zone plate.

## The conical phase profile

**Figure 2:**
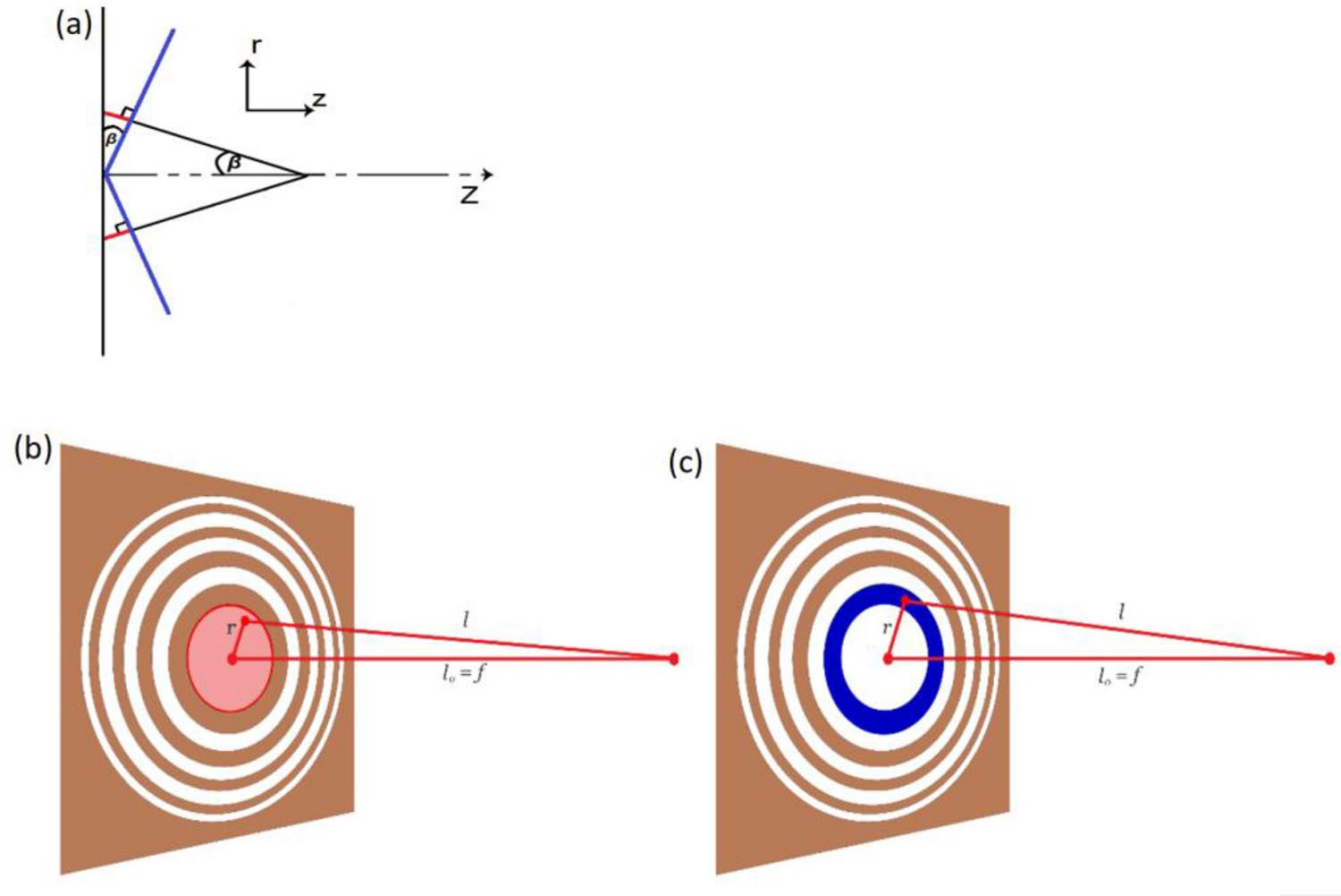
(**a**) Schematic diagram of the conical phase profile of an Axicon lens. The blue cone-like line with base angle $\upbeta$ is the required equiphase surface if we desire to design a zone plate metasurface with conical phase profile. (**b**) Schematic of the zone plate metasurface with the 1st zone shaded in red. (**c**) Schematic of the zone plate metasurface with the 2nd zone shaded in blue.

For the bulky conical Axicon lens, the phase profile would also be conical and the phase shift $\mathrm{\varphi }\left(\mathrm{r}\right)$ marked by the red segments in Fig. 2 could be given by ^32^:

1 $$\varphi \left(r\right)=k \Delta l=k \left|r\right|\mathit{sin}\beta ,$$

where $\mathrm{k}=\frac{2\pi }{\lambda }$, $\upbeta$ is an angle corresponding to the numerical aperture of the Axicon and $\Delta l$ is the optical pathlength difference represented by the red segments in Fig. 2a. The blue cone-like line is the required equiphase surface if we desire to design a zone plate metasurface with conical phase profile.

## The positions of zone boundaries in the zone plate with conical phase profile

The analysis in chapter 9 in Ref.^33^ shows how to convert a refractive spherical lens into a Fresnel-like lens and subsequently a Fresnel zone plate. We follow the same analysis procedure here but for the Axicon conical lens with a conical profile given by Eq. (1). We derive an expression for the zone plate radii $r$ of a zone plate with focal length $f$ as shown in Fig. 2b.

Assume the existence of a source point at a distance $r$ from the center of the zone plate shown in Fig. 2b where the point lies in the plane of the zone plate. For constructive interference at the focus, we require that the optical path lengths $l$ differ by no more than $\frac{\lambda }{2}$ from the optical pathlength of the on-axis source ${l}_{o}$:

2 $$l-{l}_{o}< \frac{\lambda }{2}.$$

The source points which satisfy the criterion in Eq. (2) define the 1st zone that is shaded in red in Fig. 2b. As we move away from the center of the zone plate, the quantity $l-{l}_{o}$ will increase beyond $\frac{\lambda }{2}$ where the sources in the second zone should satisfy:

3 $$\frac{\lambda }{2}<l-{l}_{o}<\lambda ,$$

where there is a destructive interference between the sources of the 2nd zone and the sources of the 1st zone creating the second zone that is shaded in blue in Fig. 2c.

By following in the same manner, the general definition of the nth zone is all the source points with optical pathlength that satisfies:

4 $$\frac{\left(m-1\right) \lambda }{2}<l-{l}_{o}<\frac{m \lambda }{2},$$

where $m$ is a positive integer ranging from 1 to M which is the total number of zones in the zone plate. Source points in even zones ($m$ = 2, 4, 6,…) make destructive interference with the 1st zone while source points in odd zones ($m$ = 1, 3, 5,…) make constructive interference. To maximize the constructive interference, we block the even numbered zones with an absorber and leave the odd zones transparent for the wave to pass through.

From Eq. (4), the boundaries between the opaque and transparent zones are defined by:

5 $$l-{l}_{o}=\frac{m \lambda }{2}.$$

From Eq. (1) of a conical phase profile, the optical pathlength difference is given by:

6 $$\Delta l=\left|r\right|\mathit{sin}\beta ,$$

where $\Delta l=l-{l}_{o}$ and thus from Eqs. (5) and (6), we arrive to the following expression for the radii ${\varvec{r}}$ of a zone plate with conical phase profile:

7 $$\left|{r}_{m}\right|=\frac{m \lambda }{2\mathit{sin}\beta },$$

where ${r}_{m}$ represents the boundary of the mth zone. To find the distance between the boundary of each zone and its neighbor’s boundary, we calculate $|{r}_{m+1}|-|{r}_{m}|$ as follows:

8 $$\Delta r=\left|{r}_{m+1}\right|-\left|{r}_{m}\right|=\frac{ \lambda }{2\mathit{sin}\beta }.$$

It is obvious that $\Delta r$ does not depend on $m$ or $f$ and thus it is uniform along the axicon zone plate lens and this is an advantage in comparison to the case of spherical zone plate lens where the radii difference $\Delta r$ depends on $m$ and decreases as $m$ increases which puts a limitation on the number of zones that could be fabricated in case of the spherical profile zone plate^33^. So the only parameters of design in case of the zone plate metasurface with conical phase profile are $\lambda$ and $\beta$. If we have a number of zones $M$, then the radius $R$ of the zone plate is given by:

9 $$R=M\Delta r.$$

## Design of three zone plates for high sensitivity refractive index sensing of methane gas at the wavelength of 3.3 μm

The analysis S1 in the supplemental document summarizes the main detailed analysis in our previous work in Ref.^30^. This analysis shows the relation that governs the variation of the axial field intensity with the change in the refractive index of the surrounding medium. This is very crucial to understand and appreciate the results in this paper.

Suppose we want to design a zone plate sensor with high sensitivity at the wavelength of 3.3 μm where at that wavelength, the absorption of methane gas is maximum and thus a maximum change in the refractive index due to this gas is expected. We have tried using three materials in our design namely soda lime glass, silicon dioxide and silicon. Starting with soda lime glass that has refractive index $n=$ 1.4789 at the design wavelength (3.3 μm). Suppose the default medium around the zone plate is air of refractive index $n{^{\prime}}=1$ then substituting those values in the following condition that we derived in Ref.^30^:

10 $${\alpha }_{cr}={sin}^{-1}\left(\frac{{n}^{^{\prime}}}{n}\right),$$

gives ${\alpha }_{cr}$= 42.54° where ${\alpha }_{cr}$ is the Axicon base angle that leads to the maximum sensitivity of refractive index sensing. It is also the critical angle at which total internal reflection happens and the rays emerge grazing to the Axicon surface. Therefore in order to maximize the sensitivity, we chose $\alpha =$ 42° which is slightly less than the critical angle ${\alpha }_{cr}.$

**Figure 3:**
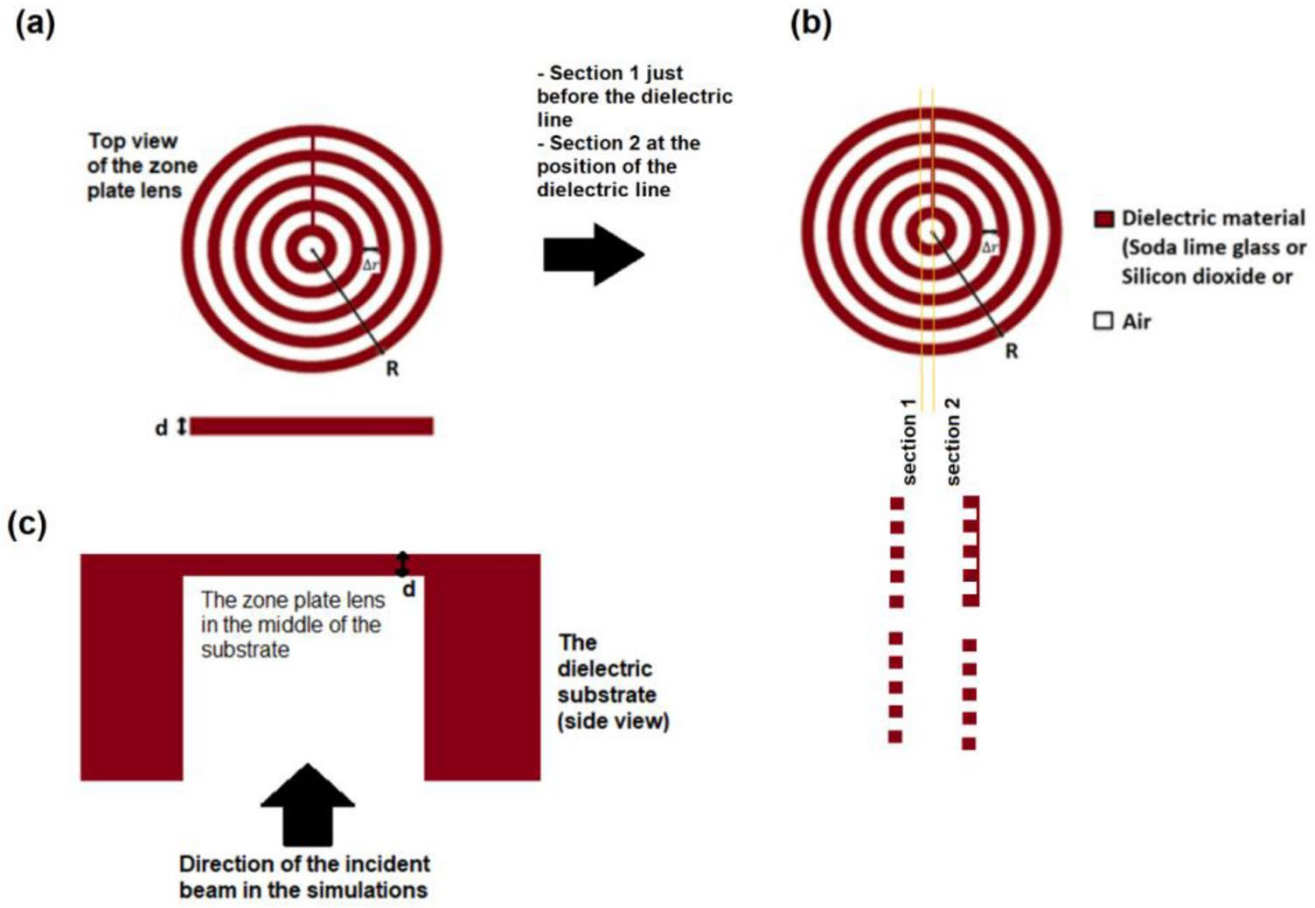
(**a**) A schematic for the top view of the zone lens and its side view of thickness d, where the dielectric material could be (soda lime glass, silicon dioxide or silicon) and the spacing between every zone boundary and its preceding or following zone boundary is uniform and equals to $\Delta \mathrm{r}$ and the radius of the whole zone plate is $\mathrm{R}$. (**b**) Two sections at the position of the dielectric line connecting the rings, and just before it. (**c**) A schematic of the simulated structure and the direction of the incident input beam in the simulations.

Substituting with $n=$ 1.4789, $n{^{\prime}}=1$ and $\alpha =$ 42° into Snell’s rule equation in Ref.^30^:

11 $$n sin\left(\alpha \right)={n}^{^{\prime}}\mathit{sin}\left(\alpha + \beta \right),$$

gives $\beta$ = 39.72° where $\beta$ is the angle the rays make with the horizontal as shown in Fig. 2a of the conical phase profile. Then, substituting this value of $\beta$ together with $\lambda$ = 3.3 μm into Eq. (8) gives $\Delta r=$ 2.58 μm. If we design our zone plate to have a number of zones $M$ = 10, then from Eq. (9), the radius of zone plate would be μm = 25.8 μm. We do the same design steps and calculations but for silicon dioxide ($n=$ 1.4133) and silicon ($n=$ 3.4335). The values $({\alpha }_{cr} , \alpha , \Delta r, M \; \mathrm{and} \; R )$ for the three lenses (Soda lime glass, silicon dioxide and silicon) are shown in Table 1. Figure 3a shows a schematic for the top view of the zone plate lens and its side view of thickness d. The spacing between every zone boundary and its preceding or following neighbour is uniform and equals to $\Delta r$ and the radius of the whole zone plate is $R$. The grooves have a depth of $d$ where $d$ was chosen to be 0.5 μm. This choice is just arbitrary. Notice the dark red line connecting the rings in Fig. 3a. This is a line of dielectric material that is suggested to connect the rings in fabrication so that they are not floating. The structure might seem like it is hanging in the air but in reality, it is not. Our structure is all slits but the slits are held together by a connecting line of dielectric material. If the structure is cut at “Introduction” as shown in Fig. 3b, it would appear like a group of floating slits. However, if the structure is cut at “From bulky Axicon to compact binary zone plate” where the dielectric line exits, it would appear as a group of floating slits in one half and as a grating in the other half (as shown in Fig. 3b).

**Table 1:** Comparison between the three zone plates (soda lime glass, silicon dioxide, silicon).

	Soda lime glass zone plate	Silicon dioxide zone plate	Silicon zone plate
Refractive index $n$ at $\lambda$ = 3.3 μm	1.4789	1.4133	3.4335
Extinction coefficient $k$ at $\lambda$ = 3.3 μm	0.00009341	0.00018	0
The critical angle ${\alpha }_{cr}$	42.54°	45.03°	16.93°
The chosen angle ${\alpha }_{design}$	42°	44°	16°
The spacing between zones $\Delta r$	2.58 μm	2.87 μm	2 μm
The number of zones $M$	10	10	10
The radius of the whole zone plate $R$	25.8 μm	28.7 μm	20 μm
Depth of grooves $d$	0.5 μm	0.5	0.5 μm
Gaussian input beam waist radius ${w}_{o}$	12 μm	14 μm	10 μm
Transmission efficiency T	93.45%	94.9%	88.6%
The full width at half maximum FWHM	5.35 μm	6 μm	3.2 μm
Focal length $f$	11.65 μm	15.25 μm	4.57 μm
Sensitivity $S$	599% per RIU	618.6% per RIU	732% per RIU

Thus the rings of the structure might seem as if they are hanging in the air, but indeed they are held together by this fine line and the substrate itself. This line can be easily made in fabrication by etching through all the slits and leaving this line not etched. Figure 3b shows the simulated structure and the direction of the incident input beam in the simulations.

**Figure 4:**
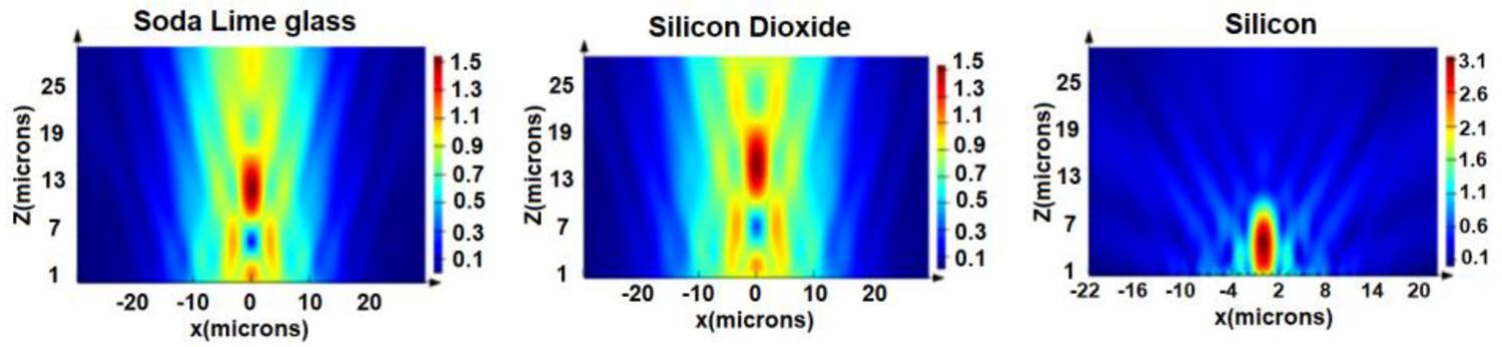
The electric field intensity distribution in the output for the three zone plates (soda lime glass, silicon dioxide and silicon).

**Figure 5:**
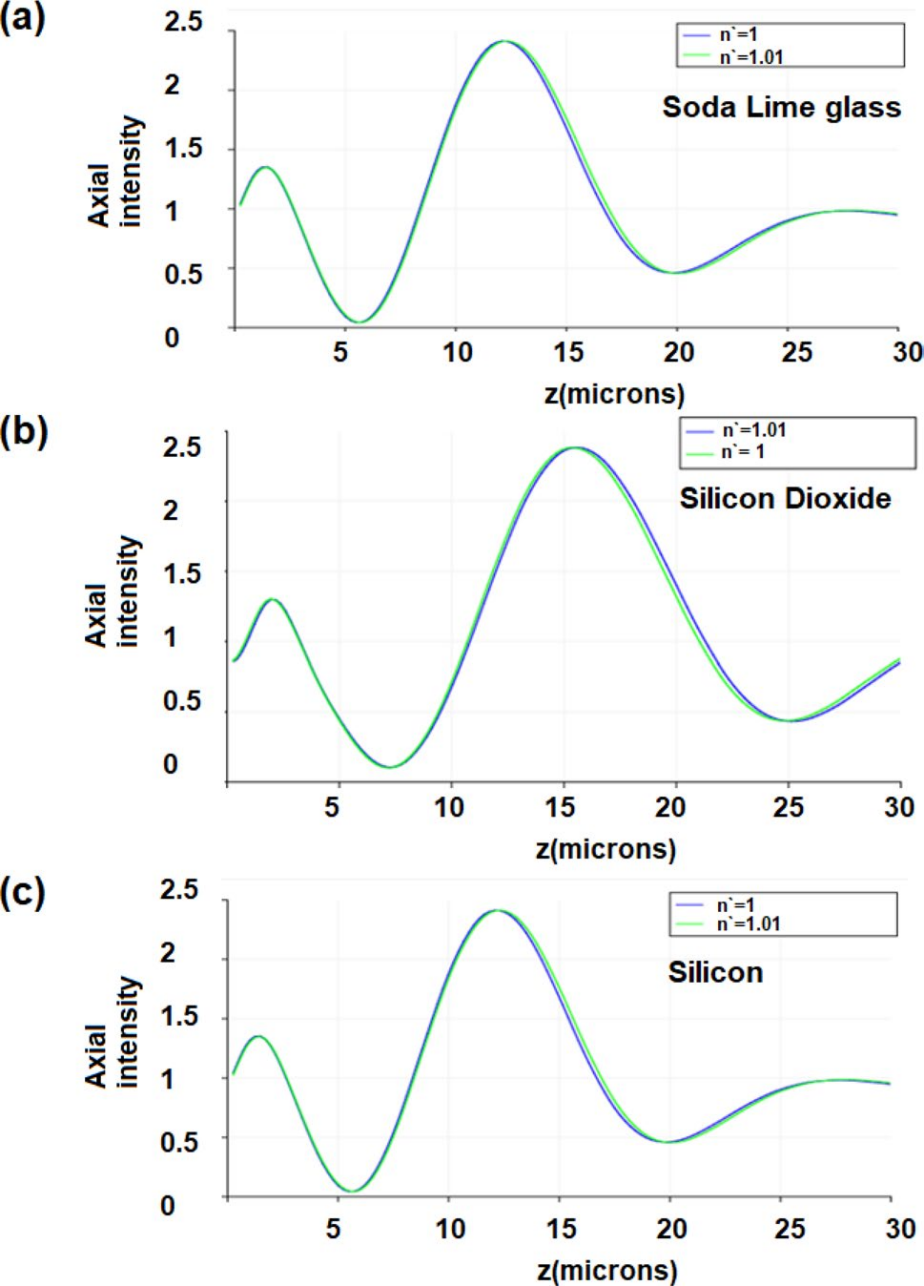
The electric field axial intensity at surrounding medium indices (n′ = 1 and n′ = 1.01) for the three zone plates (**a**) Soda lime glass, (**b**) Silicon dioxide and (**c**) Silicon.

We run FDTD simulations for the three zone plates. We used a Gaussian beam input of beam waist radius ${w}_{o}$ = 12 μm in case of the soda lime glass zone plate, ${w}_{o}$ = 14 μm in case of the silicon dioxide plate and ${w}_{o}$ = 10 μm in case of the silicon zone plate. The boundary conditions are PML layers (perfectly matched layers). Figure 4 shows the electric field distribution in the output for the three zone plates. The transmission efficiency was found to be 93.45% (for soda lime glass Zone plate), 94.9% (for silicon dioxide zone plate) and 88.6% (for silicon zone plate). In order to calculate the sensitivity of sensing for each of the three metasurfaces, we run FDTD simulations for each zone plate at different refractive indices of the surrounding medium (${n}^{^{\prime}}=1 \; \mathrm{and} \; {n}^{^{\prime}}=1.01)$ as shown in Fig. 5.

**Figure 6:**
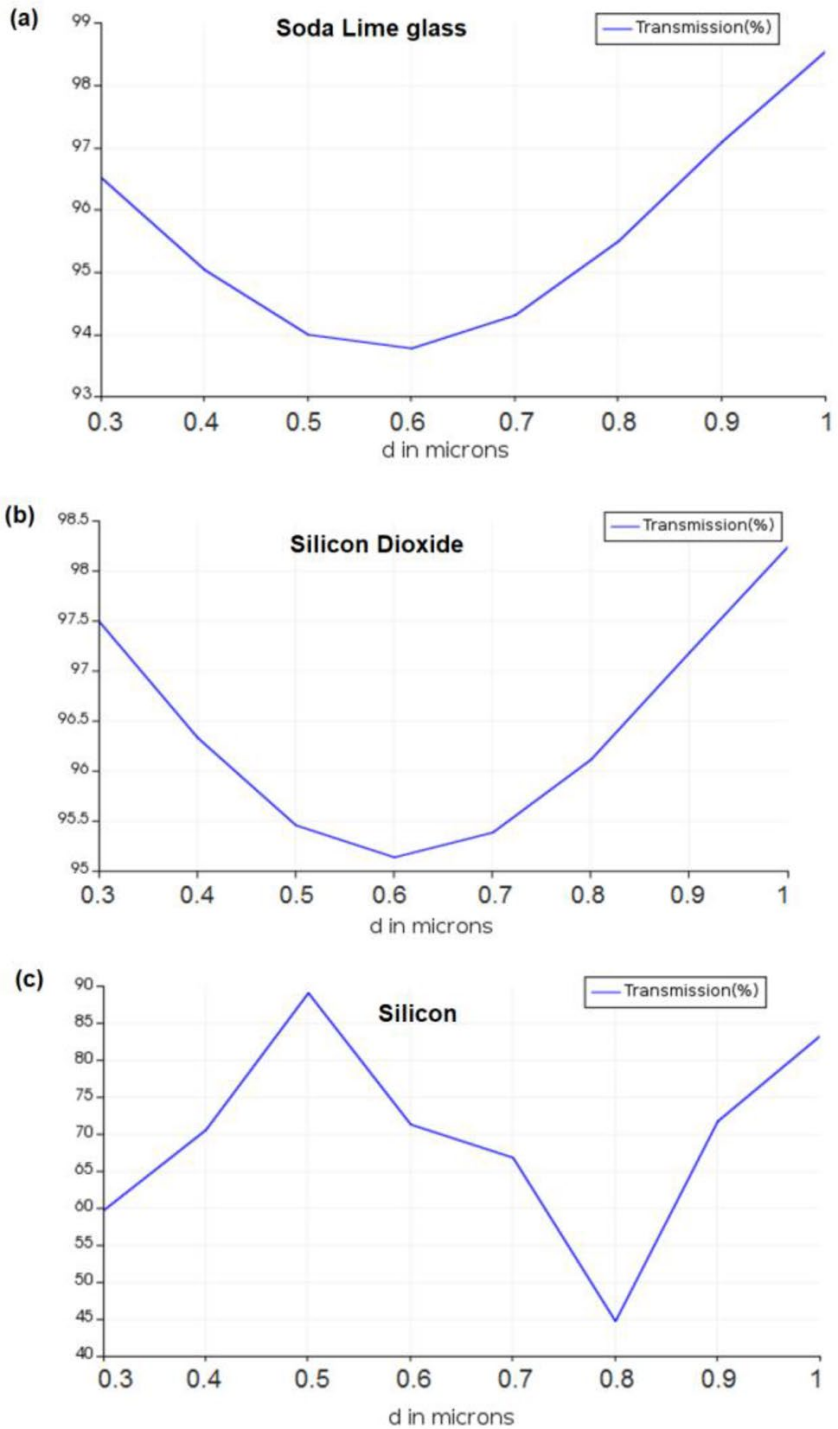
The variation of the transmission efficiency versus the variation of the zone plate thickness d in microns for the three structures (**a**) Soda lime glass, (**b**) Silicon dioxide and (**c**) Silicon.

Figure 6 shows the variation of the transmission efficiency for the three structures versus the variation of the zone plate thickness d in microns. We chose d = 0.5 μm for our structures but other values could be chosen as well and would result in different phase shifts.

We are presenting a table of comparison (Table 1) as a summary of the results and design parameters of each zone plate. It is obvious from Table 1 that the silicon zone plate has the least transmission efficiency (88.6%) and the best focusing resolution or FWHM (3.2 μm) while the silicon dioxide glass zone plate gives the highest transmission efficiency (94.9%) yet the least focusing resolution or FWHM (6 μm). The sensitivity calculated in each case is the percentage change in field intensity per unit change in refractive index (RIU).

**Figure 7:**
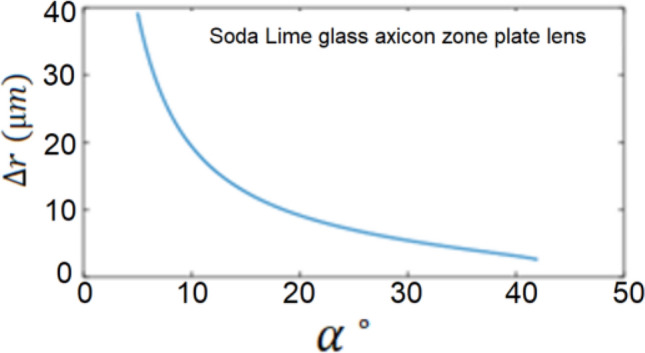
The variation of the spacing between the zones $\Delta \mathrm{r}$ with the design angle $\mathrm{\alpha }$ in case of soda lime glass material. The surrounding medium is air (${\mathrm{n}}^{\mathrm{^{\prime}}}=1)$.

Using Eqs. (8) and (11), we plot in Fig. 7 the variation of the spacing between the zones $\Delta r$ with the design angle $\alpha$ for soda lime glass material as an example. We plotted this relation for other materials and we also got the same reverse nonlinear dependency between $\Delta r$ and $\alpha$.

## Conclusion

In this work, we showed how a bulky conical Axicon lens can be converted into a zone plate metasurface which is more compact and has less dielectric and thus introduces lower losses. We derived the expression that determines the boundaries of the zone plate areas in this case and we showed how it can be designed and used for sensing of the surrounding medium index with high sensitivity that reaches 732% per RIU in case of using silicon to make the lens, 618.6% per RIU in case of silicon dioxide and 599% per RIU in case of soda lime glass. All the three zone plates operate at the wavelength of 3.3 μm at which the absorption of methane gas is maximum.

Other systems like^34,35^ use surface plasmons for sensing, but the surface plasmon systems cannot work in Mid-infrared with good performance due to the weak confinement on metal surface in the MIR range. In addition, the SPR system is bulky, costly, and cannot be utilized in a handheld system like our proposed system.

### Consent for publication

The author hereby consents to publication of the work.

## Supplementary Information

Supplementary Information.
