# Supplementary material for: High sensitivity refractive index sensing using zone plate metasurfaces with a conical phase profile
Source: Sci Rep. 2022 May 28;12:8978. doi: 10.1038/s41598-022-12849-3 (PMC9148316; doi:10.1038/s41598-022-12849-3)
Supplement: Supplementary file 1 — Supplementary Information. [file 41598_2022_12849_MOESM1_ESM.docx]

High sensitivity refractive index sensing using zone plate metasurfaces with a conical phase profile: supplemental document

Fig. S1 shows a conical axicon of radius $R_{o}$and base angle α. The angle β corresponds to the numerical aperture of the axicon and $n$ is its refractive index. An incident beam of radius $R$ strikes the lens from the left and every general ray in the beam enters the lens at a height ρ and emerges at a horizontal distance $z$ measured from the plane $z=0$ as shown in Fig. S1. The outermost ray in the beam corresponding to the beam radius $R$ emerges at a distance $z_{D}$which is the depth of focus of the lens in this case. The lens is surrounded by a medium of refractive index n' that is to be sensed.


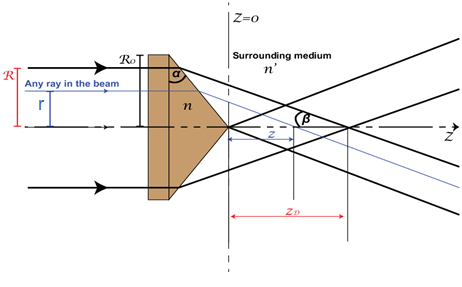


Figure S1. Refractive conical axicon lens

The following analysis is a summary of the main detailed analysis in our previous work in ref. [34] in the manuscript. The reader can seek more details by returning to the original work in [34].

**Analysis S1.** There are generally two ways we can describe the intensity profile generated from an axicon lens surrounded by air. It could be described by the scalar wave optics using the method of stationary phase [S1] which is given by:

$I\left( r,z \right)=C^{2}2\pi kI_{o}\alpha^{2} \left( n-1 \right)^{2}ze^{-2\left[ \left( n-1 \right) \frac{z \alpha}{w_{o}} \right]^{2}}{J_{o}}^{2}\left( k\left( n-1 \right)r \alpha\right)$ (S1)

where $k=\frac{2\pi}{\lambda}$, $I_{o}$ is the incident on-axis intensity, $\alpha$ is the axicon base angle, $n$ is the refractive index of the axicon lens, $z$ and $r$ are the longitudinal and radial coordinates respectively, $w_{o}$ is the incident beam waist and $C$ is a factor independent of $r$ and is given by [S1]:

$C=|C(\omega)|={|T}_{1}{\left( \omega\right) T}_{2}(\omega)exp(iknt)|=|T_{1}(\omega)T_{2}(\omega)|$ (S2)

The intensity profile of the generated Bessel-Gauss beam could also be given by [S2]:

$I\left( r,z \right)=2\pi kI_{o}{tan}^{2}\alpha\left( n-1 \right)^{2}ze^{-2\left[ \left( n-1 \right) \frac{z tan\alpha}{w_{o}} \right]^{2}}{J_{o}}^{2}\left( k\left( n-1 \right)r tan\alpha\right)$ (S3)

Both equations (S1) and (S3) are very similar to each other and they are approximate relations that follow from the following equation [S3] by assuming that $\alpha$ is a very small angle:

$I\left( r,z \right)=\frac{4\pi^{2}E^{2}(R)}{\lambda}\frac{Rsin\beta}{\cos^{2}\beta} {J_{o}}^{2}\left( \frac{2\pi r sin\beta}{\lambda} \right), R\leq R_{o} and z\leq z_{D}$ (S4)

Thus equations (S1) and (S3) would constitute a crude approximation in our case since we are trying to maximize the sensitivity by maximizing$\alpha$ and hence $\alpha$ cannot be assumed to be small in our analysis. However, equation (S3) might be a less crude approximation since it replaces $\alpha$ with $\tan\alpha$ and thus it might serve as a good qualitative estimate in our case but not an exact one because it still uses the following approximation (S5) to simplify the relation of equation (S4):

$\beta=(n-1)\alpha$ (S5)

To get the axial intensity, we put $r=0$ in equation (S3) and for a surrounding medium of refractive index $n'$, the term $\left( n-1 \right)$would be replaced with $\left( n-n' \right)$or $\left( n^{'}-n \right)$ where the order of $n^{'}$ and $n$ does not matter because of the squaring, so equation (S3) reduces to:

$I\left( 0,z \right)=2\pi kI_{o}{tan}^{2}\alpha\left( n^{'}-n \right)^{2}ze^{-2\left[ \left( n^{'}-n \right) \frac{z tan\alpha}{w_{o}} \right]^{2}}$ (S6)

We will differentiate equation (S6) with respect to $n'$ to find the sensitivity of the variation in the axial intensity as a result of the variation in the index of the surrounding medium. Differentiating equation (S6) gives:

$\frac{dI}{dn^{'}}=2\pi kI_{o}{tan}^{2}\alpha\left[ 2z\left( n^{'}-n \right)+\left( n^{'}-n \right)^{2}\frac{dz}{dn^{'}}-4\left( n^{'}-n \right)^{3}z^{3}\frac{{tan}^{2}\alpha}{{w_{o}}^{2}}-4\left( n^{'}-n \right)^{4}z^{2}\frac{{tan}^{2}\alpha}{{w_{o}}^{2}}\frac{dz}{dn^{'}} \right]e^{-2\left( \left( n^{'}-n \right) z \frac{tan\alpha}{w_{o}} \right)^{2}}$ (S7)

where $\frac{\mathrm{dz}}{dn^{'}}$ is given by the following equation derived in ref. [34]:

$\frac{dz}{dn^{'}}=\frac{dz}{d\beta} \frac{d\beta}{dn^{'}}= \frac{r}{n^{'}} \frac{\tan\left( \alpha+ \beta\right)}{{sin}^{2}\left( \beta\right)}$ (S8)

**References**

S1. Y. Wang, S. Yan, A.T. Friberg, D. Kuebel, and T. D. Visser, "Electromagnetic diffraction theory of refractive axicon lenses," J. Opt. Soc. Am. A 34, 1201-1211 (2017).

S2. I. Alexeev, K.-H. Leitz, A. Otto, and M. Schmidt, “Application of Bessel beams for ultrafast laser volume structuring of non transparent media,” Phys. Procedia 5, 533–540 (2010).

S3. R. Arimoto, C. Saloma, T. Tanaka, and S. Kawata, "Imaging properties of axicon in a scanning optical system," Appl. Opt. 31, 6653-6657 (1992).
